# Population Preferences for Performance and Explainability of Artificial Intelligence in Health Care: Choice-Based Conjoint Survey

**DOI:** 10.2196/26611

**Published:** 2021-12-13

**Authors:** Thomas Ploug, Anna Sundby, Thomas B Moeslund, Søren Holm

**Affiliations:** 1 Department of Communication and Psychology Aalborg University Copenhagen Denmark; 2 Visual Analysis and Perception Lab Aalborg University Aalborg Denmark; 3 Centre for Social Ethics and Policy University of Manchester Manchester United Kingdom

**Keywords:** artificial Intelligence, performance, transparency, explainability, population preferences, public policy

## Abstract

**Background:**

Certain types of artificial intelligence (AI), that is, deep learning models, can outperform health care professionals in particular domains. Such models hold considerable promise for improved diagnostics, treatment, and prevention, as well as more cost-efficient health care. They are, however, opaque in the sense that their exact reasoning cannot be fully explicated. Different stakeholders have emphasized the importance of the transparency/explainability of AI decision making. Transparency/explainability may come at the cost of performance. There is need for a public policy regulating the use of AI in health care that balances the societal interests in high performance as well as in transparency/explainability. A public policy should consider the wider public’s interests in such features of AI.

**Objective:**

This study elicited the public’s preferences for the performance and explainability of AI decision making in health care and determined whether these preferences depend on respondent characteristics, including trust in health and technology and fears and hopes regarding AI.

**Methods:**

We conducted a choice-based conjoint survey of public preferences for attributes of AI decision making in health care in a representative sample of the adult Danish population. Initial focus group interviews yielded 6 attributes playing a role in the respondents’ views on the use of AI decision support in health care: (1) type of AI decision, (2) level of explanation, (3) performance/accuracy, (4) responsibility for the final decision, (5) possibility of discrimination, and (6) severity of the disease to which the AI is applied. In total, 100 unique choice sets were developed using fractional factorial design. In a 12-task survey, respondents were asked about their preference for AI system use in hospitals in relation to 3 different scenarios.

**Results:**

Of the 1678 potential respondents, 1027 (61.2%) participated. The respondents consider the physician having the final responsibility for treatment decisions the most important attribute, with 46.8% of the total weight of attributes, followed by explainability of the decision (27.3%) and whether the system has been tested for discrimination (14.8%). Other factors, such as gender, age, level of education, whether respondents live rurally or in towns, respondents’ trust in health and technology, and respondents’ fears and hopes regarding AI, do not play a significant role in the majority of cases.

**Conclusions:**

The 3 factors that are most important to the public are, in descending order of importance, (1) that physicians are ultimately responsible for diagnostics and treatment planning, (2) that the AI decision support is explainable, and (3) that the AI system has been tested for discrimination. Public policy on AI system use in health care should give priority to such AI system use and ensure that patients are provided with information.

## Introduction

Recent developments in artificial intelligence (AI) hold considerable promise for promoting individual health and well-being, and societal flourishing. Taking medical imaging as an example, a recent review showed that although the diagnostic performance of deep learning models is generally equivalent to that of health care professionals, it may outperform such professionals in particular cases [1]. Better diagnosis and early detection may not only enable better treatment but also lead to more cost-effective public spending. However, the performance of AI models comes at a cost. The most successful deep learning models are opaque. The complexity of such models implies (1) that many aspects of the decision-making procedure cannot be fully explicated and scrutinized and (2) that the exact reasoning cannot be replicated step-by-step in real time [2]. Across all types of AI, it seems that at the moment, the better-performing models (eg, deep learning models) are the most opaque [2-8].

The opacity of a medical AI system may concern different groups in the clinical setting. Thus, there are partly dissociable transparency and explainability problems in relation to health care professionals and in relation to patients. If an AI system and its outputs can be made transparent to health care professionals, then patients can potentially rely on the trust they have in those professionals, even if the patients themselves do not understand the system. However, if there is also a significant transparency and explainability problem in relation to health care professionals, both patients and professionals end up in a situation in which the only possible reliance on trust will be on trust in the system. This paper primarily investigates this as seen from a patient perspective.

Opacity is undesirable. The transparency and explainability of AI decision making to the patient are important for psychological, scientific, ethical, and democratic reasons. Psychologically, transparency may increase the understanding of the AI process and may make it easier to cope with AI decisions significantly impacting individual lives (eg, receiving a life-changing diagnosis). Scientifically, transparency may provide insights into hitherto unknown correlations constitutive or suggestive of causal mechanisms. Ethically, transparency may provide a basis for individual self-protection against biased and discriminatory decisions, decisions based on violations of privacy, decisions subjecting individuals to unreasonable risks of harm, etc. Democratically, transparency may unveil the inner workings of the technology used in specific settings by the state or other powerful actors to exercise power over citizens, and thus, it may empower citizens to hold decision makers accountable through the institutions of democracy.

The importance of transparency and explainability of AI is widely recognized. Researchers and research institutions, public committees, and expert groups, as well as private companies, have in recent years issued guidelines for responsible use of AI, emphasizing the value of transparency. A recent systematic review found 84 such guidelines [9]. The authors showed a remarkable global convergence on the importance of transparency, but they also exhibited a significant variation in what transparency is taken to be and requires. The importance of transparency is recognized in the European Union General Data Protection Regulation (GDPR), which in Articles 13 and 14 stipulates that if data subjects are profiled, they have a right to “meaningful information about the logic involved” [10]. A right to “meaningful information” is, however, rather vague. It may be interpreted minimally as simply requiring abstract and generic information about AI involvement in decision making along the lines of “this decision was partly based on recommendations made by an automated computer system.” It may also be interpreted maximally as requiring access to all aspects of the AI decision making and the ability to reproduce each and every (significant) step in the decision making. As noted above, a maximal interpretation would entail that some of the best-performing systems of AI cannot satisfy the requirement of transparency.

The need for transparency is also recognized by researchers and developers of AI systems, and “explainable AI” is an active research field, and it is probably unlikely that any system would be implemented in health care without some work having been done estimating the importance of features such as gender, age, and ethnicity on the outputs of the system. This is, however, still not full transparent or explainable.

This paper proceeds from the assumption that there is a real dilemma here. Maximal transparency of AI systems may come at the cost of system performance and vice versa. This is not a conceptually necessary dilemma. New AI architectures may be invented that satisfy all relevant criteria of transparency and explainability and at the same time perform better than current architectures. However, until that happens, there is a balance to be struck between transparency and system performance. We believe that an adequate requirement of transparency should consider individuals’ interests and preferences for performance and transparency. We therefore studied the relative importance to citizens of these and other aspects of AI decision making in health care in the Danish population by performing a conjoint analysis survey.

## Methods

### Initial Focus Groups

In this study, 2 focus group interviews were conducted with 5-6 participants in each drawn from Kantar Gallup´s Danish consumer panel. The participants were a cross section of the public and ranged from age 27 to 75 years. Both interviews were conducted in September 2019, and each interview lasted about 2 hours. The participants were briefly introduced to AI and were subsequently presented with 2 scenarios revolving around the use of AI for decision making in health care. They were asked to discuss each of the scenarios and in particular (1) the importance of being provided with explanations of the AI decision making and (2) the trade-off between the accuracy and performance of AI decision making and being provided with explanations of the decision making. The groups were asked, for instance, how important it is to explain how AI reaches decisions, even if the ability to do so will make the AI decisions less accurate.

All interviews were audio-recorded and transcribed verbatim. The interviews in combination with the literature offered information that was used to identify 6 aspects that play a role in the participants’ views on the use of AI in health care: (1) type of AI decision (ie, whether it is used for diagnostics or treatment planning), (2) the level of explanation available, (3) performance and accuracy, (4) responsibility for the final decision, (5) possibility of bias or discrimination, and (6) severity of the disease or condition to which the AI is applied.

### Design of Survey

**Figure 1:**
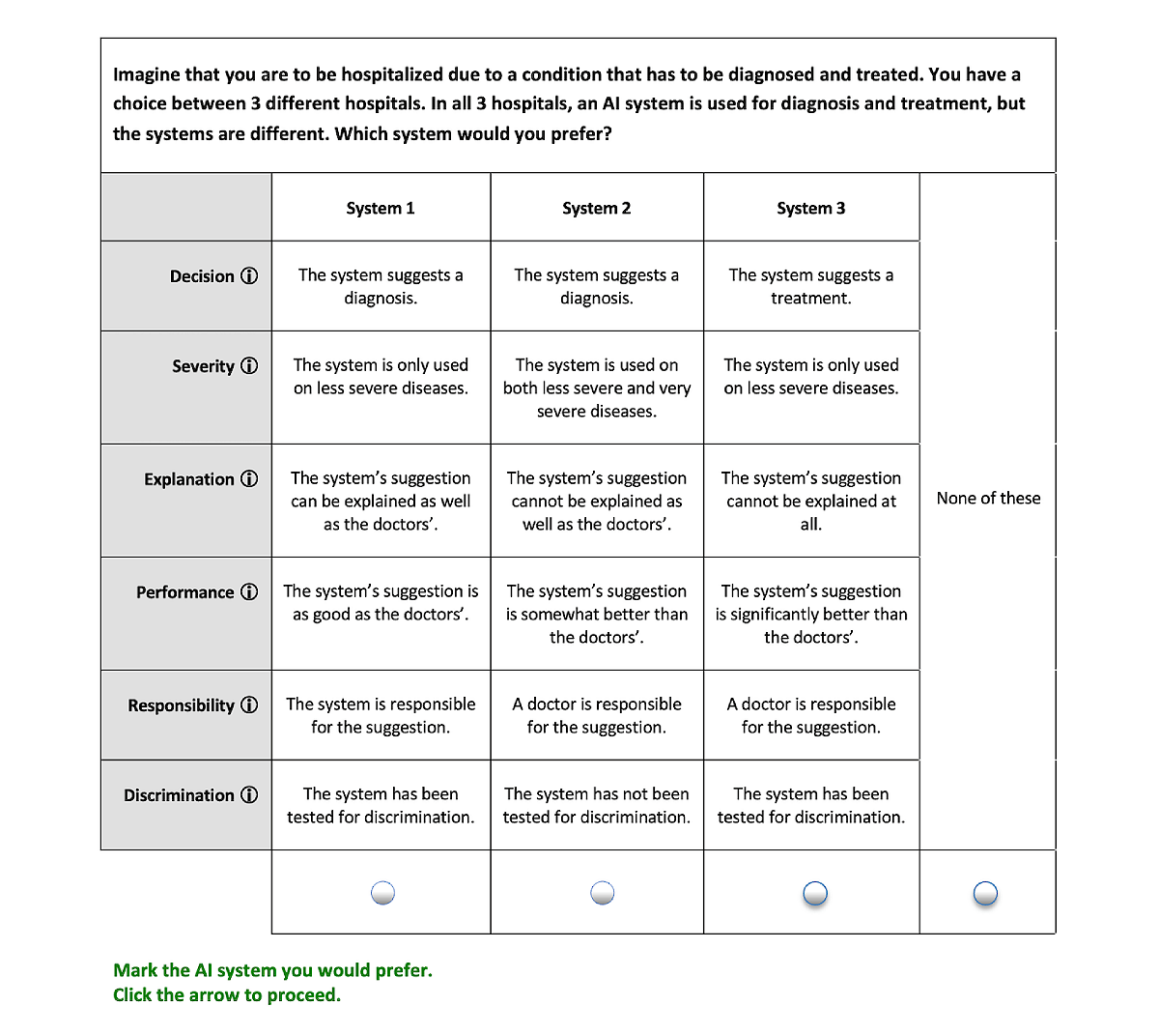
An example of a choice task with 3 concepts. AI: artificial intelligence.

Conjoint analysis is a discrete choice survey methodology. Respondents are asked to make a choice between 2 or more different options, where each option is described in terms of a number of predefined attributes, each with a number of levels (Figure 1). Given a sufficient number of choices per respondent, it is then possible to statistically estimate the importance of each attribute and level for the choice in terms of part-worth utilities [11].

The 6 aspects mentioned above were chosen as attributes for the conjoint analysis survey. For each attribute, a number of levels were developed based on the literature. In setting the lowest level for performance, it was assumed that any AI system introduced in health care would be known to perform at least as well as a trained health care professional (see also the Discussion section later).

The choice sets were generated using the complete enumeration method in the Sawtooth SSI Web (version 7.0.30) module [12]. The complete enumeration method generates conjoint designs conforming to the principles of (1) minimal overlap of attribute levels within a single choice task, (2) level balance across the set of choice tasks presented to each respondent, and (3) orthogonality (ie, the levels of different attributes are chosen independently). In total, 100 unique sets of conjoint choice questionnaires were generated, each of which was presented to an approximately equal number of respondents. Each set of conjoint choice questionnaires contained 12 choice tasks, where each respondent was asked to choose 1 of 3 options or a “None of these” option.

The choice situation was described as follows:

*This study is about artificial intelligence (AI). AI is a way of getting digital technologies to solve complex tasks. There is, for instance, AI in self-driving cars, search engines on the web, or the voice assistants in mobile phones. AI is often based on large data sources. You can, for instance, train an AI system to make diagnoses of cataract or melanoma by showing it many different pictures of eyes or skin with moles. You can also train AI systems to make suggestions about treatment of a disease (eg, suggestions for medication). AI systems have shown themselves to be quite good at making diagnoses and suggestions about treatment. However, they also sometimes make errors or differentiate unjustly between patients. In addition, it can be difficult to explain how a diagnosis or a suggestion for treatment has been derived. In what follows, you will therefore be asked about what you would prioritize if an AI system was used in the health care sector to make a diagnosis or suggest a treatment for you. On the next 12-15 pages, you will be shown 3 different scenarios that all involve the use of AI for diagnosis and treatment. On each page, you should choose the scenario that you think is best/you prefer. If none of the scenarios look good to you, there is an option “None of these.”*

In addition to the conjoint analysis survey, respondents were asked about demographic data, chronic illness, and recent contact with the health care system; questions about trust in the health care system; and questions about fear and hope in relation to AI in general [13].

### Sample

A stratified sample of 1678 potential participants was drawn from Kantar Gallup’s Danish consumer panel of 53,000 active members. The sample was designed to be representative of the adult Danish population. Emails were sent to the potential participants, inviting them to participate in the study. After 3, 11, and 29 days, nonresponders (ie, those who had not completed the survey or who had not visited the website hosting the survey) were reminded by email. After 6, 22, and 31 days, nonresponders were contacted by SMS.

Of the 1678 potential respondents contacted, 1441 opened the link to the questionnaire, 1027 completed it fully, and 414 completed it partially. The analysis was based on the 1027 complete answers, giving a response rate of 61.2% (1027/1678). A sample efficiency analysis calculating the overall concordance between the respondents and the desired sample characteristics was performed, considering obtained and desired numbers in relation to gender, age, geographical region, and level of education (sum of squares=33.78, *df*=785, efficiency=88.33%).

### Statistical Analysis

The analysis of the conjoint analysis survey deriving the part-worth utilities of the attributes and levels was performed by Kantar Gallup. Part-worth utilities were estimated using Sawtooth CBC/HB (version 5.5.3) to perform a hierarchical Bayes method estimation, running 190,000 burn-in iterations and 10,000 draws per respondent. A detailed description of the hierarchical Bayes method and its implementation can be found in Ref. [14].

The subsequent statistical analysis was performed using IBM SPSS Statistics 25. Demographic data were tabulated and univariate relationships between utilities and respondent characteristics analyzed using ANOVA with Bonferroni correction. Ad hoc trust, fear, and hope scales were formed as simple summative scales from the trust, fear, and hope questions and validated by Cronbach α. All 3 scales had acceptable α values of .74-.79. Univariate relationships between utilities and the 3 scales were analyzed using correlation analysis with Bonferroni correction.

## Results

### Major Findings

A total of 521 of 1027 (50.7%) respondents were men and 506 (49.3%) women. The average age was 50.3 years (SD 18.1). Of the 1027 respondents, 375 (36.5%) indicated that they had a chronic illness, 830 (80.8%) had visited their general practitioner (GP) at least once during the past year, and 146 (14.2%) had been inpatients in a hospital during the past year. The highest educational level was school or high school for 197 of 1027 (19.2%) respondents, further education for 446 of 1027 (43.4%) respondents, and university or university college for 384 of 1027 (37.4%) respondents.

The part-worth utilities of the attributes and levels are presented in Table 1, the responses to questions about trust in health care and technology, and fear and hope in relation to AI in general in Table 2, and the relationship between respondent characteristics and utilities of attributes in Table 3.

**Table 1:** Importance of attributes and part-worth utilities of levels.

Attribute	Importance (%)	Level (part-worth utility)
Type	3.0	Diagnostics (0.123) Treatment planning (–0.123)
Explanation	27.3	Equally explainable as physician’s decision (1.106) Not as explainable as physician’s decision (–0.270) No explanation available (–0.836)
Performance	6.6	System decision significantly better than physician’s (0.267) System decision somewhat better than physician’s (0.052) System decision equally good as physician’s (–0.319)
Responsibility	46.8	Physician responsible for decision (1.900) System responsible for decision (–1.900)
Discrimination	14.8	System tested for biased decisions (0.602) System not been tested for biased decisions (–0.602)
Severity of disease	1.5	System use only when less severe disease (0.060) System use both when less severe and when very severe disease (–0.060)

**Table 2:** Respondent trust and opinions about AI^a^ (N=1027).

Opinion		None/not at all, n (%)	Very little, n (%)	Little, n (%)	Some, n (%)	A lot/certainly, n (%)	Don’t know, n (%)
**Trust**							
	I have trust in the health care system.	7 (0.7)	35 (3.4)	102 (9.9)	438 (42.6)	424 (41.3)	21 (2.1)
	I have trust in physicians.	2 (0.2)	29 (2.8)	72 (7.0)	412 (40.1)	502 (48.9)	10 (1.0)
	I have trust in technology.	4 (0.4)	32 (3.1)	129 (12.6)	519 (50.5)	313 (30.5)	30 (2.9)
**Fear**							
	I believe that AI will lead to unemployment.	122 (11.9)	216 (21.0)	251 (24.4)	169 (16.5)	95 (9.3)	174 (16.9)
	I believe that AI will cause unintentional harm to humans.	55 (5.4)	206 (20.1)	303 (29.5)	181 (17.6)	67 (6.5)	215 (20.9)
	I believe that AI will lead to loss of control to machines.	86 (8.4)	167 (16.3)	249 (24.2)	241 (23.5)	141 (13.7)	143 (13.9)
	I believe that AI will lead to increased data collection and mass surveillance.	22 (2.1)	30 (2.9)	106 (10.3)	309 (30.1)	435 (42.4)	125 (12.2)
**Hope**							
	I believe that AI will lead to more jobs.	119 (11.6)	180 (17.5)	300 (29.2)	164 (16.0)	55 (5.3)	209 (20.4)
	I believe that AI will lead to longer lives.	69 (6.7)	114 (11.1)	243 (23.6)	284 (27.7)	92 (9.0)	225 (21.9)
	I believe that AI will lead to more quality of life.	82 (8.0)	119 (11.6)	279 (27.2)	265 (25.8)	93 (9.0)	189 (18.4)
	I believe that AI will lead to peace and political stability.	225 (21.9)	209 (20.4)	232 (22.6)	66 (6.4)	20 (1.9)	275 (26.8)

^a^AI: artificial intelligence.

**Table 3:** Respondent characteristics and the importance of attributes.^a^

Attribute (average weight)	Gender	Age	Level of education	Urban/rural background	Chronic disease	Inpatient last year	GP^b^ visits last year	Trust scale	Fear scale	Hope scale
Type (0.12268)	—^c^	—	—	—	—	—	—	—	—	*P*=.002 r=–.097
Explanation (1.10638)	—	—	—	—	—	—	—	—	—	—
Performance (0.31895)	*P*=.01 M^d^=.337 F^e^=.300	*P*<.001 More impor­tant with lower age	*P*<.001 Lowest level of education=.179 Highest level of education=.659	*P*=.002 Most rural=.271 Most urban=.354	—	—	—	*P*=.003 r=.093	*P*<.001 r=.170	*P*<.001 r=.243
Responsibility (1.90018)	—	—	—	*P*=.01 Most rural=1.940 Most urban=1.792	—	—	—	—	—	—
Discrimination (0.60190)	*P*<.001 M=.542 F=.682	—	—	—	—	—	—	—	—	*P*<.001 r=–.120
Severity of disease (0.06042)	—	—	*P*=.01 Lowest level of education=.122 Highest level of education=–.226	—	—	—	—	*P*=.002 r=–.099	—	*P*<.001 r=–.168

^a^Numerical data only shown for cells where there is a statistically significant difference.

^b^GP: general practitioner.

^c^Not applicable.

^d^M: male.

^e^F: female.

The results in Table 1 show that the physician having the final responsibility for treatment choice is the most important attribute, with 46.8% of the total weight being allocated to it, followed by explainability of the decision (27.3%) and whether the AI system has been tested for discrimination (14.8%). These 3 attributes accounted for 88.9% of the total weight/importance.

As can be seen in Table 2, the respondents in general trusted health care and technology; did not particularly fear AI, although some did; and in general believed that AI will have positive implications for society.

Table 3 shows that while gender, age, level of education, whether respondents live rurally or in towns, their trust in health and technology, and fear and hope regarding AI did influence the importance they allocated to different attributes, they did not play a significant role in the majority of cases. The data shown for the numerical differences between groups and the correlation coefficients indicate that the statistically significant findings do not reflect large numerical differences or strong correlations.

## Discussion

### Principal Findings

The results of this study are interesting. First, the study shows that among the respondents, there was a clear order of preference between AI performance and AI explainabillity. Being provided with an explanation was the second-most important factor (27.3%) for the respondents’ choice of preferred AI system, while performance carried little weight (6.6%) and was ranked only fourth out of the 6 attributes. However, the study also shows that the population finds a number of different aspects of AI decisions important for their choice of preferred AI system use. It is not simply a matter of choosing between the performance and explainability of AI. The single-most important factor for their choice is how responsibility for a diagnosis or treatment plan is distributed between physician and AI system. The respondents placed significant emphasis on physicians being responsible for health care decisions (46.8%).

The relatively limited role of AI system performance in the respondents’ preferences arguably reflects the chosen levels of the performance attribute. We did not in this study include the possibility that the AI system performs worse or significantly worse than physicians. This would likely have changed the overall weight of performance in the respondents’ decisions. Our design was based on what we believe to be the most likely future scenario for the implementation of AI in health care, and this does not include the introduction of AI systems that perform significantly worse than physicians. Implementing such suboptimal systems is likely to be resisted by health care professionals and will in some jurisdictions also be open to legal challenge. Our study specifically shows that in health care implementations with AI systems performing at least as well as physicians, the role of AI system performance in the populations’ preferences is limited. They are not particularly interested in getting increased performance if this leads to a loss of other important features of the AI system. On a more speculative note, the choice of a nonnumeric description of the standard of performance (“equally good,” “somewhat better,” and “significantly better”) may be thought to be less informative than providing, for instance, the accuracy of the AI system and physicians as a percentage of correct decisions made, false positives, false negatives, etc. However, providing the information about accuracy in percentage terms may be difficult to understand and may communicate a false sense of precision in our evaluation of how well a system works when implemented in a routine health care setting.

Interpreting the respondents’ strong preference for the explainability of AI decisions in health care is difficult. The explainability attribute is stated in terms of the degree of explainability relative to the explanation provided by a physician, and this entails that little can be said about what kind of explanation the respondents want or how they understand explainability. It may be reasonable to assume, however, that this standard would lead the respondents to expect limits to how fine-grained explanations of AI decision making can be made available—just as there are limits to how fine-grained explanations physicians can provide. Thus, providing patients with information about each and every aspect of diagnostics or treatment planning is not the standard of everyday clinical practice for a number of different reasons, including limits to the amount of medical information patients may be able to process and time constraints on the physician-patient encounter. We believe that the relative standard of explainability is the simplest and most meaningful way of introducing different levels of explainability of AI decision making to the respondents.

There are a number of statistically significant findings of relationships between respondent characteristics and the weight given to particular attributes (Table 3). However, when considering the size of the differences in weighting between the highest and the lowest group, it is evident that although these differences are statistically significant, they do not signify a fundamental change in the ranking of the different attributes. The negative finding that there are no significant relationships between the degree of contact with the health service and the weight given to the different attributes is also important since it indicates that there is, broadly speaking, no major differences between patients and nonpatients in their general preferences in relation to AI in health care.

Table 3 contains 2 further key findings. First, that hope in the future benefits of AI certainly is a driver of respondents’ views as to the importance of the attributes. The performance of the AI system is of greater importance for the hopeful, whereas testing for discrimination and distinguishing between the use of AI systems for diagnostics or treatment of less severe or more severe diseases is of lesser importance. Second, that the respondents’ views concerning the importance of performance are influenced by a number of factors. It is significantly more important for the younger, the educated, the urban, and respondents with fear or hope concerning a future with AI.

### Strengths and Limitations of This Study

The response rate of 61.2% is good for a population survey, and the sample efficiency analysis shows that the respondents were similar to the complete sample in relation to the stratification variables. There is therefore reason to believe that the findings reflect the views of the wider Danish population.

The choice situation is hypothetical in 2 ways: (1) The respondents are not in an actual situation demanding a choice between AI systems, and (2) currently, the level of use of AI systems for diagnostic and treatment-planning purposes in Danish health care is not as advanced as the scenarios suggest. The first of these abstractions is a feature of the conjoint choice methodology, and the second is a feature of the current level of penetration of AI in health care. The choice situation is, however, close to a clinical situation of which many respondents will have actual experience. For the purpose of deciding a general policy of transparency of AI health care decision making, we believe it is important to know the wider population’s preferences as abstracted from the distress of a real-life choice situation. A general policy should, however, consider the diversity of views and preferences of the general population, including those of patients. Of the 1027 respondents in this study, 375 (36.5%) indicated that they have a chronic disease, 146 (14.2%) had been hospitalized within the past year, and 830 (80.8%) had visited their GP within the past year. Most of the respondents are thus in regular contact with the health care system and are used to being involved in decision making in that context.

Given the rapid developments in AI over the past decade, the general population’s familiarity with the potential and actual use of AI systems for diagnostics and treatment planning may be expected to be low. Even those who realize that the voice recognition functionality in their phone or tablet relies on AI processing may not transfer that to the health care context. Asking for views and preferences in relation to hypothetical implementations of AI in hospitals therefore is likely to reflect more general views and preferences in relation to the implementation of “a new technology” in health care. These views and preferences may or may not change as the familiarity with the technology grows. Potential changes in views and preferences must be monitored and considered in an ongoing adjustment of policies. Such changes do not, however, obviate the need for policy decisions at the current stage of AI development and use in health care.

This entails that it is important that the choice situation be described in a way that does not introduce overt bias, especially in relation to the features of AI that are the attributes of the options in the choice task. There are 2 elements of our choice task that could potentially introduce bias. The first, which we discussed above, is that the performance of AI is compared to the performance of a physician and that choices therefore to some extent depend on the respondents’ perception of the typical performance of physicians. The second is our description of the transparency and performance of AI systems. We wrote, “AI systems have shown themselves to be quite good at making diagnoses and suggestions about treatment. However, they also sometimes make errors or differentiate unjustly between patients. In addition, it can be difficult to explain how a diagnosis or a suggestion for treatment has been derived.” This is a true description of current and near-future AI systems in health care and is not overtly biased. As all short descriptions, it can be made longer and complete in various ways, but putative proponents or opponents of AI in health care are likely to look for different additions and likely to point to different real-world examples, for instance, in relation to the risk of bias and discrimination. We cannot rule out bias, partly because there is no yardstick for a “neutral description,” but we would argue that the risk of bias is low.

This study was conducted in the Danish population and may not be representative of other populations. Further studies are required.

### Previous Empirical Research

A number of studies on the broader public’s perceptions of automated decision making and AI as such have been conducted in recent years [15-18]. Of particular interest is a recent study on the relative weight of 3 AI features (performance, explainability, and the effort required for implementation/training) for industry experts’ choice of a preferred AI decision support system for high-stake maintenance of airplane turbines. The study found that performance is, by far, the most important factor (0.61), whereas explainability (0.20) and effort (0.19) are on a par [19]. Although the study explores the relative weight of AI system features for decisions of a group of experts, it does not report the wider public’s perceptions. Moreover, it includes a limited set of features, and it is outside the health care context studied in this article.

Studies of perceptions of AI use in the medical context have taken different approaches. Some have studied the perceptions of AI use among health care professionals [20-23]. We focused here, however, on the public or patient perceptions of AI in the health care context. Overall, the studies on public or patient perceptions report a strong confidence in and acceptability of AI system health care use in the diagnostic context [24-30]. However, most studies also find that the respondents have higher confidence in physician diagnostics and prefer implementations of AI in medical care, with AI playing a decision-supportive role [24,25,27,28]. Several studies indicate that respondents have a strong interest in the performance or accuracy of AI diagnostic systems [28,31]. One study reports a marked difference in respondents’ confidence between AI use for diagnostic and treatment decision purposes, with a significant lower confidence in the latter [25]. A study of factors driving the perceived risks of AI clinical device use concludes that perceived uncertainties about performance, concerns about potentially reduced communication with physicians, perceived untrustworthiness of AI, perceived lack of regulatory standards for evaluating the safety and impact of AI, and concerns about liability issues are significant drivers of the perceived risk [31]. Notably, the study also finds that privacy concerns and concerns about social biases and discrimination do not significantly impact the perceived risk of AI clinical device use [31]. Although these previous studies concern perceptions of several features of AI health care use, none of them investigate public perceptions of the importance of AI explainability and perceptions of the relative weight of features of AI decision making in health care. Most of the findings are, however, compatible with our findings.

### Policy Implications: The Use of AI in Health Care and Requirements of Transparency

Deciding issues of public policy cannot be done entirely on the basis of individual or population preferences. Various other ethical, legal, professional, and political concerns must be considered. There are, however, at least 2 reasons for considering individual and population preferences. First, the principle of respecting and promoting individual autonomy is usually taken to entail that it counts in favor of an action or a policy if it provides individuals with an opportunity to protect and pursue their interests. Second, the ideal of representative democracy is usually taken to entail that in deciding public policies, decision makers should represent the interests of the people. Designing public policy partly on the basis of population preferences, as mapped in this conjoint analysis survey, is a way of considering individuals’ interest at an aggregate level.

This study is of relevance for policies concerning the implementation of AI in health care. The study suggests that AI systems will be found acceptable and can be used for both diagnostic and treatment-planning purposes regardless of the severity of the medical condition if the system has been tested for discrimination, if decisions can be explained to the same extent as physicians’ health care decisions, if the physicians are ultimately responsible for the health care decisions, and if the performance of the AI system is at least as good as that of physicians.

The study also has some implications for the question of the transparency of AI decision making in health care. The study clearly shows that a requirement of transparency cannot be dismissed on the grounds that in the eyes of the population, performance is the only significant concern. On the contrary, the population not only takes explainability to be considerably more important than performance, but it also puts significant emphasis on other aspects of AI decision making. The mere fact that all aspects of the use of AI were considered somewhat important by the respondents could be taken to support the view that the transparency of AI decision making in health care should concern more than the narrow explainability of the AI decision. Transparency should also be a matter of providing patients with information about responsibility and testing for discrimination, performance, and the character of the use of AI.

Taking a more comprehensive approach to transparency fits recent writings on the ethics of transparency of AI use in health care. Thus, it has been argued that transparency in relation to AI-based diagnostics and treatment planning is a matter of providing patients with information that will enable them to effectively contest these decisions [32]. This includes information not only about the key indicators behind an AI-generated diagnosis or treatment plan but also about the performance of AI, bias testing, and the distribution of responsibility between physicians and AI systems.

### Conclusion

This paper proceeded from the assumption that AI system performance and AI explainability/transparency are potentially in conflict. Currently, the decision making of the best-performing deep learning models cannot be fully scrutinized or replicated step-by-step. We believe this tension must be resolved in and through appropriate policy making, and we have argued here that an appropriate policy should consider the population’s interests in and views concerning AI system features, such as performance and explainability/transparency. The findings of the choice-based conjoint survey reported in this paper are that if an AI diagnostic system does not perform any worse than a physician, then the 3 factors that are most important to the public are, in descending order of importance, (1) that physicians are ultimately responsible for diagnostics and treatment planning, (2) that the AI decision support is explainable, and (3) that the AI system has been tested for discrimination. A policy of AI system use in the health care setting should give priority to AI systems that can meet these requirements and should provide patients with information about the division of labor and responsibility between physicians and the AI system, the key explanatory factors in the AI system decision making, and the bias testing of the AI system. However, this study links the notion of AI explainability to the explainability of physicians’ decision making. How AI system explainability can be achieved in a way that makes it relevantly similar to physician decision making is an obvious avenue for further research.

## Abbreviations

AI: artificial intelligence
GDPR: General Data Protection Regulation
GP: general practitioner
